# Springback effect and structural features during the drying of silica aerogels tracked by in-situ synchrotron X-ray scattering

**DOI:** 10.1038/s41598-022-11127-6

**Published:** 2022-05-09

**Authors:** Fabian Zemke, Ernesto Scoppola, Ulla Simon, Maged F. Bekheet, Wolfgang Wagermaier, Aleksander Gurlo

**Affiliations:** 1 Technische Universität Berlin, Faculty III Process Sciences, Institute of Materials Science and Technology, Chair of Advanced Ceramic Materials, Straße des 17. Juni 135, 10623 Berlin, Germany; 2 Max Planck Institute of Colloids and Interfaces, Department of Biomaterials, Am Mühlenberg 1, 14476 Potsdam, Germany

**Keywords:** Characterization and analytical techniques, Design, synthesis and processing, Nanoscale materials

## Abstract

The springback effect during ambient pressure drying of aerogels is an interesting structural phenomenon, consisting of a severe shrinkage followed by almost complete re-expansion. The drying of gels causes shrinkage, whereas re-expansion is believed to be linked to repelling forces on the nanoscale. A multi-scale structural characterization of this significant volume change is key in controlling aerogel processing and properties. In this work, hydrophobic, monolithic silica aerogels with high specific surface areas were synthesized by modification with trimethylchlorosilane and ambient pressure drying. Here, we report a multi-method approach focusing on in-situ X-ray scattering to observe alterations of the nanostructured material during the drying of surface-modified and unmodified silica gels. Both show a porous fractal nanostructure, which partially collapses during drying and only recovers in surface-modified samples during the springback effect. Distinct changes of the X-ray scattering data were reproducibly associated with the shrinkage, re-expansion and drying of the gel network. Our findings may contribute to tailor aerogels with specific functionality, as the springback effect has a direct influence on properties (e.g., porosity, pore size distribution), which is directly affected by the degree of re-expansion.

## Introduction

Because of their remarkable properties, such as a low density, very high specific surface area and porosity, low thermal conductivity, and low dielectric constant, aerogels are promising candidates for several applications^1^. They are highly porous structures, typically synthesized by a sol–gel process and afterwards dried without significant volume change^2^. Though aerogels can be produced from gels of various materials, silica remains one of the most studied systems^3^. Silica aerogels are usually synthesized by supercritical drying (SCD) through diligent solvent exchange and drying procedures. An alternative approach is the so-called ambient pressure drying (APD), which does not require supercritical conditions. However, the latter approach applies only to materials with a proper surface modification^3^. A reason for that is a so-called springback effect (SBE) that is usually observed during aerogel processing with APD, in which the gel undergoes severe shrinkage and re-expansion. In some cases, materials can even shrink by up to 50% and recover as far as 95% of their original volume^4,5^. For a ceramic (i.e., inorganic, non-metallic) material^6^, this reversibility of shrinkage is surprising. Deficient expansion progress might lead to altered properties or cracking of the ceramic material. APD-proceeded gels that do not undergo the SBE, i.e., do not recover their volume after drying, exhibit higher densities and lower porosities and may not be called aerogels^2^.

During APD, liquid evaporation from the gel network creates compressive stress on the solid phase, which results in shrinkage of the network. Hydrophobization of the material grants reversibility of the volume change^7^. Hydrophobic endgroups can be introduced by surface modification of the gel using silylating agents, such as trimethylchlorosilane (TMCS) or hexamethyldisilazane (HMDZ)^8^. Although different aerogel-like monoliths can be obtained by ambient pressure drying, re-expansion of the material has only been reported for materials where non-polar endgroups were introduced. Whereas, strengthening the network or minimizing the capillary pressure induced by the evaporation of the solvent might reduce the overall volume shrinkage of the material, the SBE may only occur with sufficient hydrophobization of the network^5,9,10^.

Insufficient hydrophobization leads to condensation reactions of silanol groups and irreversible shrinkage of the network^11^. The SBE is believed to occur because of the viscoelastic behavior of hydrophobized gels, which is influenced by the density and aging of the material^12^. The latter increases the stiffness of the network by densification, and when done insufficiently, might lead to a pore collapse^13^. The aging can control partly the strength and flexibility of the gel structure, which is of importance to withstand the capillary pressures and the re-expansion^14,15^.

The sol–gel synthesis and formation of the gel network are often discussed in terms of hydrolysis and condensation reactions^16^, while the aerogel formation might also be influenced by mechanical stresses and related changes in the nanostructure. The mechanical stiffness on the nanoscale is of increased importance, since the SBE and densification are governed by it^15^.

According to previous studies, the nanostructure of silica aerogels can be described as a network of interconnected chains of weakly branched spherical particles (3–100 nm) with intermediate pores (5–100 nm) in between and a fractal geometry or randomly packed colloidal aggregates^17^. The sizes and structures of particles, clusters, and pores of this aggregated gel can be researched by means of small-angle X-ray scattering (SAXS)^18^, providing insight into the aggregation behavior during formation and the interfacial roughness between the two phases^19^. Parameters describing the nanostructure can be related to the intensity decay of SAXS profiles in specific reciprocal space regions such as fractal and Porod regions^20^. The first one can be assigned to a three-dimensional branched network and a mass fractal with self-similarity i.e., a structural or mass-related similarity across several length scales^21^, while the second one is usually associated with surface fractals and interfacial roughness^22^. For example, significant changes in the fractal and Porod regions were found for wet and dry gels, and correlated to different network features^23^. Furthermore, a change in the fractal region was attributed to the aging of aerogels^24^.

In this work, we report a SAXS characterization over the complete drying process, both in-situ and ex-situ, of ambient pressure-dried silica aerogels. We have succeeded in correlating the SBE with structural changes both in the network of particles as well as pores in aerogels during the drying and evaporation of hexane used as a solvent. Both the pure silica and surface-modified silica by TMCS silylating agent were investigated. Since the former neither displays any hydrophobicity nor possesses the SBE^5^, the comparison of pure and functionalized silica allows for the identification of SAXS fingerprints that can be associated with the structural features in both samples. Understanding the changes during the SBE is inevitable to control the ambient pressure drying process of aerogels.

## Results and discussion

**Figure 1:**
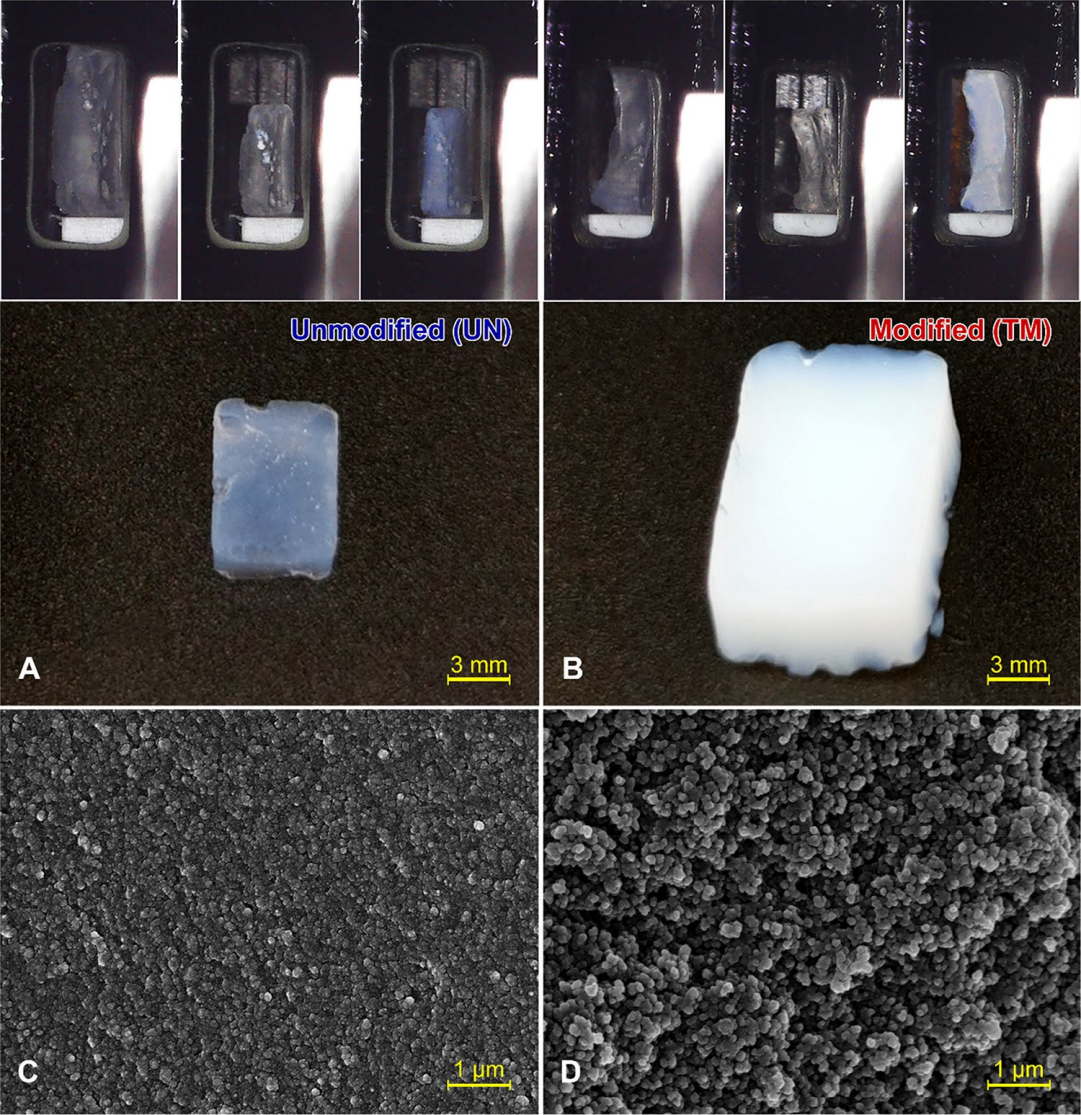
(**A**,**B**) Photographs of dried unmodified UN and modified TM samples, showing a different width, color, and transparency. Additionally, distinct changes while drying and the shrinkage of the samples are shown above. (**C**,**D**) SEM images of an unmodified UN and modified TM sample, displaying severely different surface porosity at the micrometer scale.

In a first characterization step, ex-situ measurements were performed on both, unmodified (UN) and TMCS surface modified (TM) silica samples dried for five days. The samples were compared by means of optical imaging (Fig. 1A,B) and electron microscopy (Fig. 1C,D). The dried samples show a different geometry, color, and transparency. While the width of the UN sample was approximately 6 mm, the TM sample appeared to expand fully to the original size with a width of 10 mm. While both specimens had the typical blueish color due to Rayleigh scattering^25^, the UN sample was transparent in comparison to the translucent TM sample.

Noticeable changes in the microscopical structure due to surface modification were ascertained by scanning electron microscopy (SEM) investigations. The UN sample (Fig. 1C) exhibits microscopically dense structures, which seem to consist of packed, smaller units. In contrast, the TM sample shows a high porosity (Fig. 1D) with a structure of interconnected chains of sphere-like units. This difference in network particle size can be attributed to the recovery of the structure for the TM sample, in contrast to the densification of the UN sample. These results suggest that the surface modification influences the overall porosity of the material and the pore network structure.

**Figure 2:**
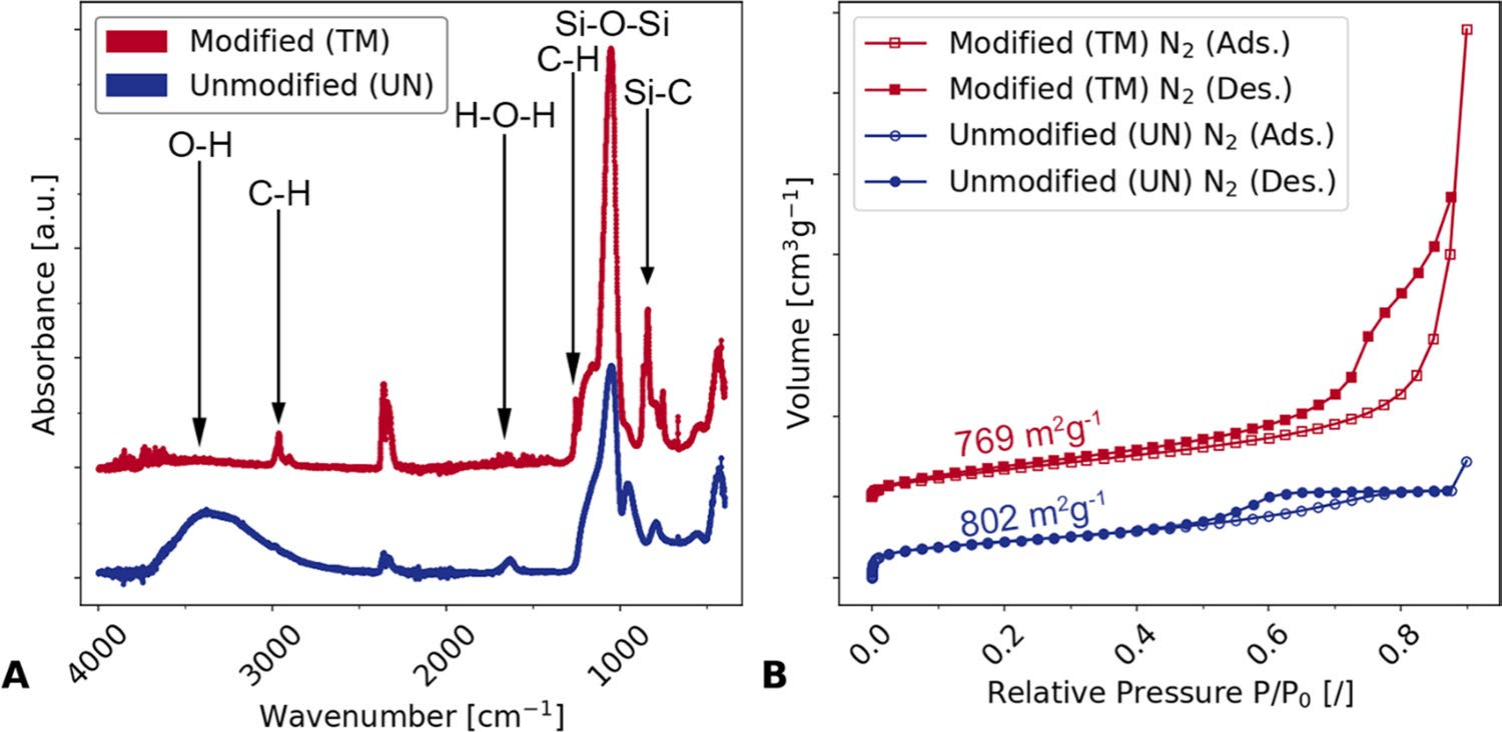
(**A**) ATR-FTIR of the TMCS surface-modified TM (red) and the unmodified UN (blue) dried sol–gel samples confirm the successful modification of the gel surface with TMCS. The TM sample is shifted by a Y-offset. (**B**) Nitrogen adsorption (Ads.)—desorption (Des.) isotherms of the dried UN (blue) and the y-offset shifted TM (red) sample show similarities for the specific surface area evaluation after Brunauer–Emmet–Teller (BET) and severely different volumes at high relative pressure.

Structural changes due to surface modification by TMCS were characterized by means of ATR-FTIR spectroscopy (Fig. 2A) of the UN and TM samples. Some relevant differences were found: A broad absorption band corresponding to O–H group (3500 cm^−1^) was observed for UN but not for the TM sample. The same difference was recorded at a lower wavenumber (1630 cm^−1^), where a less pronounced peak corresponding to H–O–H vibration, indicative of adsorbed water, appeared for the UN but not for the TM sample. On the contrary, absorption bands corresponding to C–H (2900 cm^−1^, 1310 cm^−1^) and Si–C (840 cm^−1^) were observed for the TM and not for the UN sample. Additionally, FTIR displays a Si–O–Si vibration (1100 cm^−1^) for both samples, typical for the silica network structure^26^. These results prove the surface modification by TMCS, as this silylation agent reacts with the terminal hydroxy groups to form hydrophobic Si–O–Si(CH_3_)_3_ and hydrochloric acid^27^, which was removed by solvent exchange with hexane prior to drying.

The nitrogen sorption measurements (Fig. 2B, see also Figure S13) and evaluations of the specific surface area according to the Brunauer–Emmet–Teller (BET) method reveal specific surface areas of 769 m^2^g^−1^ and 802 m^2^g^−1^ for the TM and UN samples, respectively. The specific surface area of the unmodified sample was slightly higher than the surface-modified sample, which was already reported in the literature and was attributed to pore blocking and loss in surface roughness with the modification agent^28^. Furthermore, the nitrogen sorption data revealed a typical Type IV(a) adsorption–desorption isotherm with hysteresis, characteristic for mesoporous materials^29^. Although both samples showed a steep rise of the isotherms at low relative pressures indicative of the presence of micropores, the UN sample increased further, thus suggesting a higher amount of micropores. Additionally, the hysteresis of the TM sample increased up to a relative pressure of 0.9, suggesting a high contribution of large mesopores and small macropores, which is in correspondence with the SEM images.

**Figure 3:**
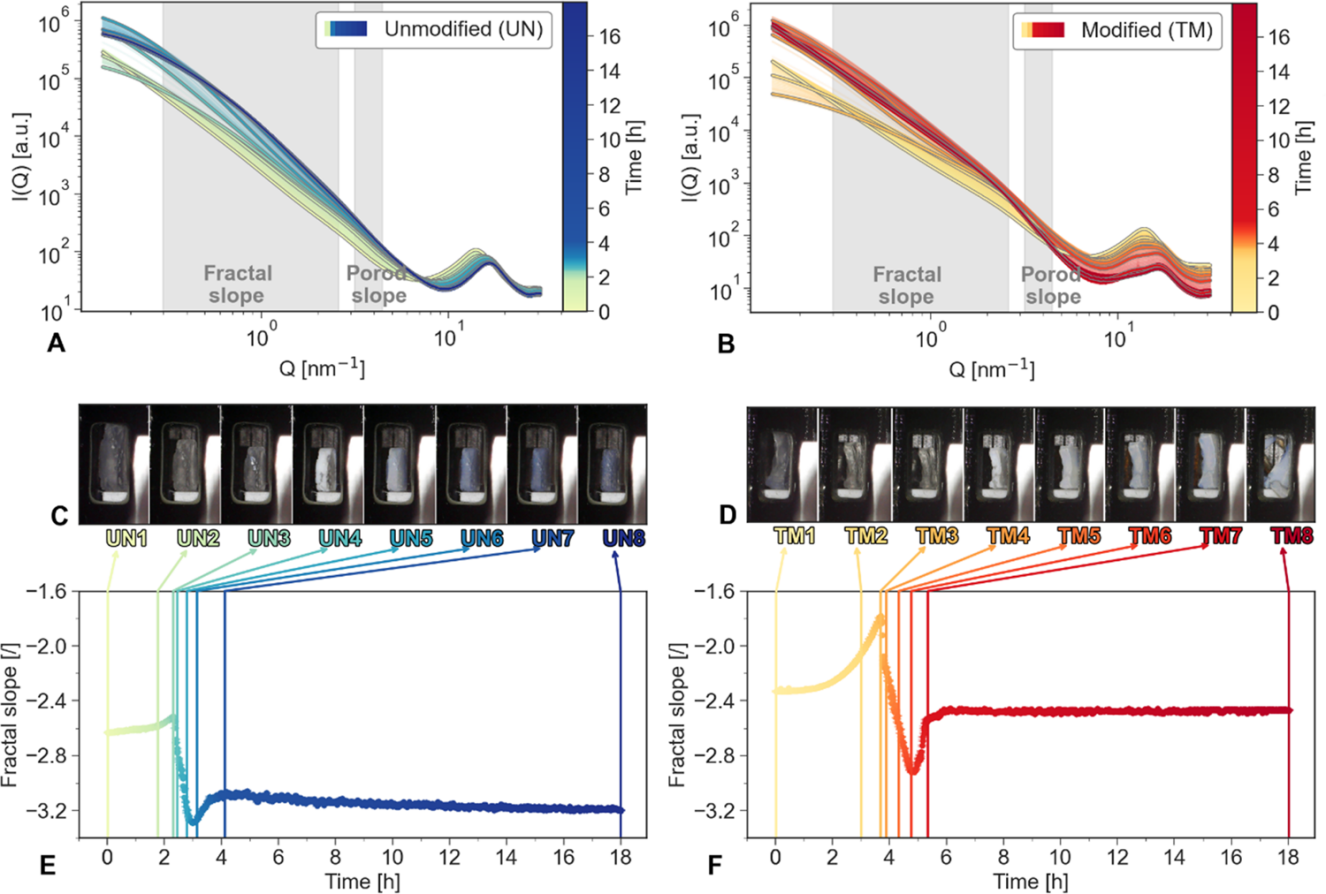
(**A**,**B**) Small-angle X-ray scattering (SAXS) diagrams of the unmodified UN (green/blue) and modified TM (yellow/red) samples over the duration of the drying. Specific measurements are highlighted by a grey stroke. (**C**,**D**) Selected photographs according to noticeable changes in fractal slope for both samples UN and TM. (**E**,**F**) Slope fits for the fractal slope for the duration of the experiment of UN (green/blue) and TM (yellow/red) samples.

In-situ small/wide-angle X-ray scattering (SAXS/WAXS) experiments were performed to record structural changes of wet gels during the drying process and understand the nanoscopic manifestation of the SBE. To this end, cuboid-shaped samples were successfully prepared and stored in an excess amount of hexane. The scattering profiles measured during the SAXS experiments are shown in Fig. 3A,B for the UN and the TM samples, respectively (see also Figure S12). Even though the absolute intensity was different, the shape and decay of scattering profiles of the TM sample at the end and start of the experiment were similar, suggesting a similar form but different volume fractions of constituents’ particles that build the network structure. Similar decay but with higher intensity is attributed to the change in the electron density, while moving from a 2-phase system of silica/hexane to a 3-phase system of silica/hexane/air to finally a 2-phase system of silica/air. The corresponding electron density contrast evolution caused by differences between hexane and air is negligible when analysing the SAXS profiles. In fact, for a given pore/solid volume ratio, hexane replacement by air would increase the pore/solid electron density difference, and subsequently, the scattered intensity, but does not affect the characteristics of the profile. These observations can be attributed to the re-expansion caused by the SBE, meaning that the large pores collapsed during drying and recovered upon SBE. Contrary, the scattering profiles of the UN sample did not recover their original shape, and consequently, this sample regained neither its original nanostructure nor its macroscopic shape. Further studies probing lower $Q$ regions might be useful to observe cluster size changes^18^.

With increasing drying time, noticeable differences at $Q$ < 0.4 nm^−1^ occurred for UN and TM samples, where both samples exhibited a flattening of the curve until a pronounced plateau was visible. At this point, the samples demonstrated many similarities in this $Q$ region. In a later stage of the drying process, the shape of the scattering curve of the UN sample did not change, whereas the TM specimen developed a lesser pronounced flattening, and recovered the linear decay.

To elucidate changes in fractal characteristics of the samples during drying, SAXS profiles were analysed in an intermediate *Q* range ($0.3 {\mathrm{nm}}^{-1} \le Q \le 2.6 {\mathrm{nm}}^{-1}$). During the experiment, the change of electron density contrast might affect the *Q* position of this fractal region, which was attributed for by allowing for a shift in the fitted *Q* range (see Note S1). The corresponding slope values of the UN and TM sample, here called “fractal slope”, as a function of time are displayed in Fig. 3E,F, respectively. For both samples, the fractal slope evolution was used to set distinct points of interest (e.g., maximum and minimum), denoted as UN1–UN8 and TM1-TM8 and correlated with macroscopic volume changes estimated by measuring an area inside images collected by a digital microscope camera (Fig. 3C,D, see also Figure S10 and Figure S11).

At first, an initial increase of the fractal slope was associated with a severe shrinkage of approximately 45% of the original sample size (UN1 to UN3 and TM1 to TM3). The TM sample started with a fractal slope of − 2.33 ± 0.01 (TM1) and raised to − 1.79 ± 0.01 (TM3), whereas the UN sample increased only slightly, ranging from roughly − 2.63 ± 0.01 (UN1) to − 2.52 ± 0.01 (UN3). Afterwards, a sharp decrease of the fractal slope (− 2.57 ± 0.01 for TM5, − 3.20 ± 0.01 for UN5) was correlated with a change in transparency (Fig. 3C,D). The samples turned opaque-white at the edges until the whole samples lost their transparency (UN5/TM5) in approximately half an hour (TM 2288 s and UN 1757s). Shortly after, the TM sample started to expand, reaching approximately the same size (TM7) as TM1 and turning opaque-blue, while demonstrating a monotonous increase in fractal slope (− 2.54 ± 0.01 for TM7). Thereafter, no changes in size could be observed until the end of the measurements (TM8). Likewise, the fractal slope showed only a negligible decrease (− 2.47 ± 0.01 for TM8) over the duration of ca. 13 h. Contrary, the UN sample showed an additional change in colour, turning blue-transparent (UN6) and remaining in its condensed form until the measurement end (UN8). These visual changes of a transparent shrinking gel, to an opaque, and finally a blue-transparent material, are also reported in the literature for polymethylsilsesquioxane gels^30^. Here, the fractal slope of UN (− 3.09 ± 0.01 for UN7) exhibited a smaller increase than the modified sample (TM7). Similarly, a small decrease (− 3.20 ± 0.02 for UN8) was visible for the UN sample for the remaining roughly 14 h of the measurements. The maximum shrinkage was similar for TM and UN samples, suggesting no dependency of the surface modification on the material. The blue hue is assumed to arise from Rayleigh scattering of the light with the porous networks.

After the maximum shrinkage, the UN sample increased only slightly in fractal slope without recovering the initial slope, while the TM sample regained roughly the same starting values before and after drying. Thus, it is assumed that recovering the initial values of the fractal slope is a distinct feature of complete SBE. Overall, the absolute fractal slope values ranged from 1.79 to 3.27. According to Sinkó et al., a range of 2 to 3 can be correlated with mass fractals in aerogels^20^. As visualized in Fig. 3B, the TM sample demonstrates a linear decay over at least one decade at the end of the measurements (TM8), allowing to associate the corresponding fractal slope (Fig. 3F) with a fractal dimension of ca. − 2.5. The latter is consistent with Witten-Sander diffusion-limited monomer-cluster aggregation^31,32^, a computer model used to predict and describe several aggregation behaviors (e.g., gelation)^33^. This approach could be used to build a structural model of the nanoscopic constituents of aerogels corresponding to their fractal dimension^34^.

**Figure 4:**
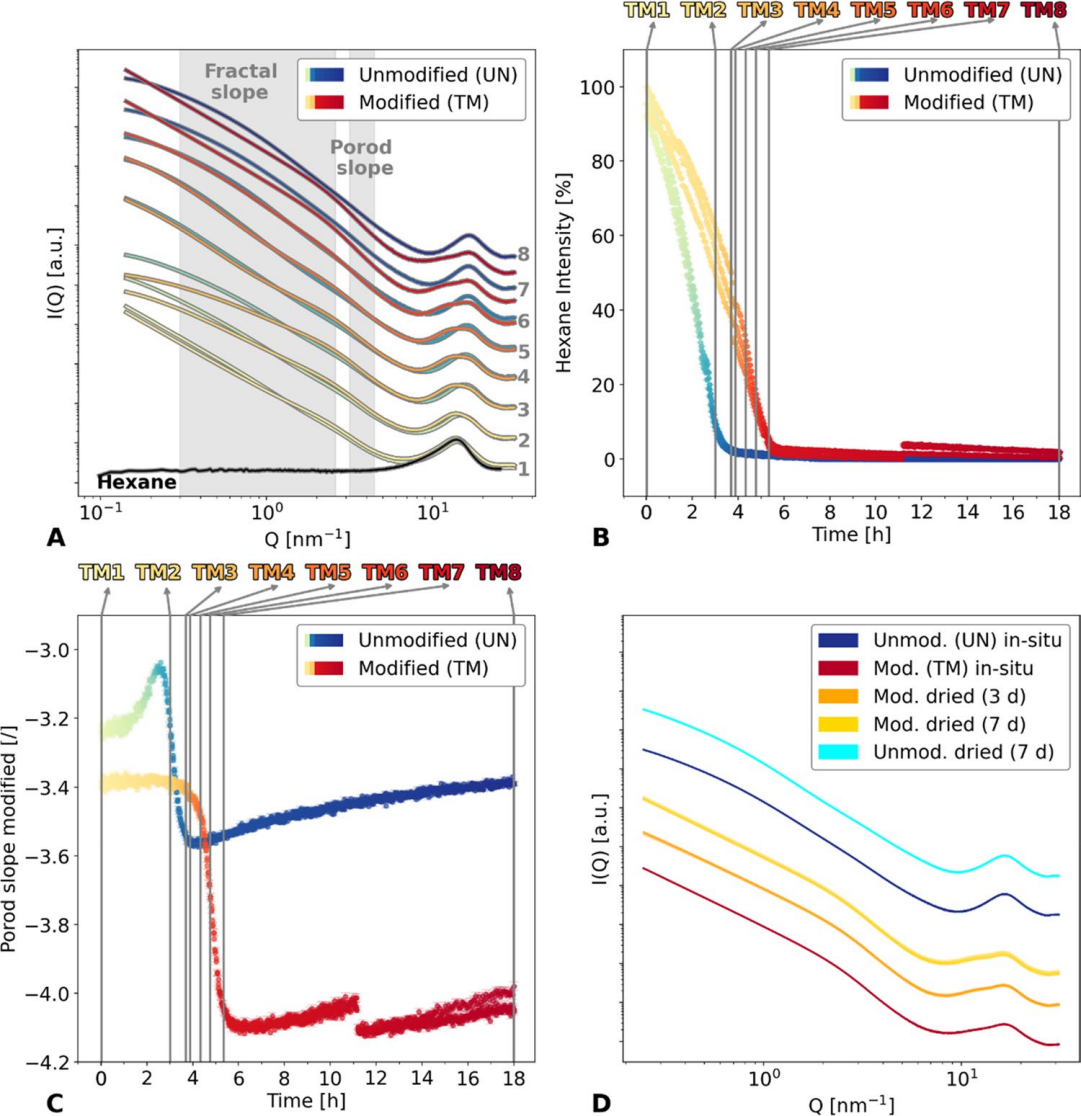
(**A**) Small-angle X-ray scattering (SAXS) diagram with Y-offset of specific points of interest (UN1 to UN8 and TM1 to TM8) are colour-coded to the appropriate time. These points of interest have been chosen according to noticeable changes of the fractal slope. (**B**) The hexane content in the UN (green/blue) and TM (yellow/red) samples over time have been derived from the decoupling of the peak in the region 13.73 nm^−1^. A split into three lines can be observed especially for TM, as the samples were investigated at three different heights. Additionally, specific points of interest are overlaid for TM1 to TM8 (grey). (**C**) The Porod slope is corrected for hexane in the material for UN (green/blue) and TM (yellow/red) samples over time, and superimposed with specific points of interest (grey, TM1 to TM8). (**D**) Different modified and unmodified samples in the dried state are compared by means of X-ray scattering. The abbreviation “TM/UN dried” refers to samples of the same batch but dried prior to the synchrotron experiments.

The findings above underline the structural evolution of the specimen can be related to the hexane evaporation process. In fact, as visualized in Fig. 3A,B, both samples show a peak around $Q=13.73$ nm^−1^ that can be attributed to the hexane, which allows an estimation of the course of hexane evaporation. An independent measurement of hexane scattering was performed in a glass capillary during a separate experiment. Figure 4A provides extracted scattering data of the distinct points of interest for both samples with the superimposed hexane scattering. While drying, the hexane peak became weaker, and eventually, a peak around $Q=16.80$ nm^−1^ became more distinct, which is likely attributed to silica. By assuming that the measured scattering profiles $I\left(Q\right)$ are a combination of the scattering originated by the specimen, ${I}_{s}\left(Q\right)$, and by a residual fraction $\alpha$ of the hexane scattering signal ${I}_{h}(Q)$, we estimated the amount hexane evaporation over time (see Note S2). We can summarize this in the following equation:

1 $$I\left(Q,t\right)={I}_{s}\left(Q,t\right)+\alpha \left(t\right)\cdot {I}_{h}\left(Q\right).$$

If at the time $t={t}_{0}=0$ the hexane content was highest, and at the end of the experiment ($t={t}_{f}$) the samples were completely dried, we can extrapolate the residual amount of hexane $\alpha$ by inverting Eq. (1).

The TM sample displayed a longer drying process over six hours as well as a non-linear drying, whereas the UN sample showed an almost linear decrease of hexane volume over the duration of three hours (Fig. 4B, see also Figure S9). Since the samples were measured at different heights, three lines with similar developments were noticeable.

Since the shrinkage of the material is governed by the capillary forces generated by the liquid/gas phase boundary inside the small pores^11^, it seems reasonable to suppose complete hexane evaporation at the point where the material was completely shrunk. As shown in Fig. 3C,D in relation to Fig. 4B, this was not the case for the samples we studied, i.e., at the time of maximal shrinkage, the hexane content was about 35 and 42 vol% of their initial amount in the UN3 and TM3 specimens, respectively.

Both samples shrunk to almost the same size, suggesting a maximal possible shrinkage for the material systems under study. As suggested in the literature, the stiffness of the gel network increases during the compression until the strength of the structure might compensate for the capillary forces^35^.

The drying velocity of the two samples shown in Fig. 4B was severely different, with ca. 7 vol% residual hexane at roughly three and five hours left for UN (6.50 ± 0.05 vol% at 10,068 s) and TM (7.01 ± 0.06 vol% at 18,756 s), respectively. This difference could be explained either by entrapment of the hexane in the pores or differences in the capillary forces in the samples.

The UN sample dried linearly and shrunk faster than the TM sample, so that porosity decreased, too, since the pores collapsed during shrinkage. This should, in return, decelerate the drying velocity, as at a given point in time before maximum shrinkage, the UN sample exhibited a smaller pore volume than the TM sample. This suggests that porosity had a negligible influence on the drying velocity. Therefore, shrinkage and porosity reduction cannot be fully correlated. Furthermore, entrapment of hexane inside the shrinking silica network can be excluded, as the internal macroscopical structure of UN and TM samples should be similar before drying, despite the surface modification. Since the TM sample was shrinking slower than the UN sample, it should have dried faster, which was not the case.

Bisson et al state that drying is roughly divided into two separate phases, namely evaporation of the fluid on the surface of the material, followed by capillary flow and diffusion. For simplification, they assumed that the wet gel could be described as a network of interconnected cylindrical pores^11^. The shrinkage during the evaporative drying is related to capillary pressures and is mainly influenced by the interfacial tension of the liquid vapour interface, the pore radius, as well as the wettability of the silica network by the solvent, which can be influenced by surface modification^28^. Accordingly, the wettability is reported to have an effect on the drying velocity^36^. Therefore, different hydrophobization and hexane affinity might be the cause of different drying velocities. Furthermore, the surface modification might block micropores, which in turn hinders the drying of the TM sample.

**Figure 5:**
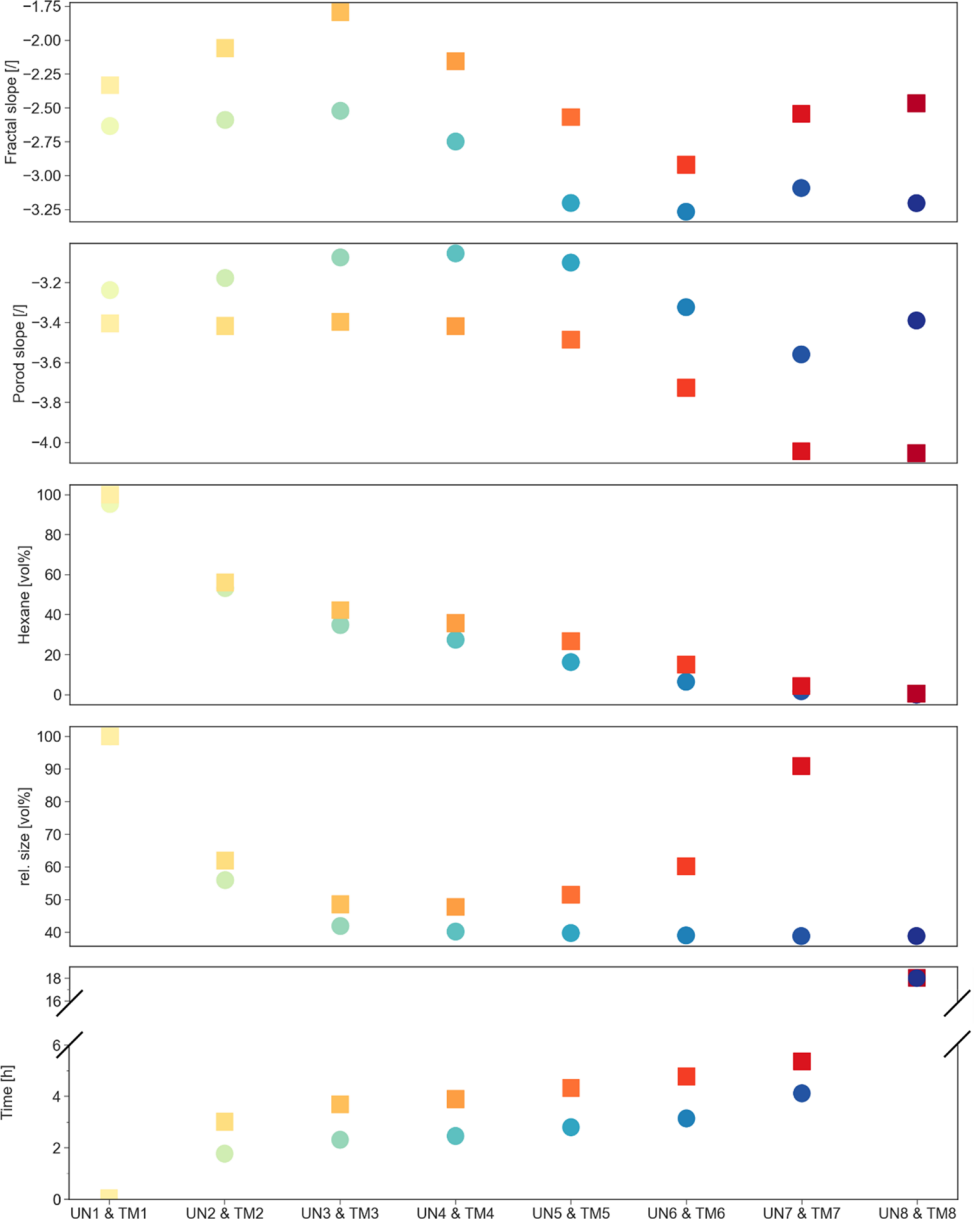
Overview of distinct features of the in-situ X-ray scattering experiment are visualized for specific points of interest UN1-UN8 (green/blue) and TM1-TM8 (yellow/red) as a function of fractal slope, Porod slope, hexane, relative size, and time, respectively. The relative size was estimated by measuring the area in the photographs. The last value (TM8) for the TM sample could not be measured due to the cracking induced by the sample falling over.

Information about the pore and material interfacial roughness at the nanoscale can be obtained by a linear fitting of the double logarithmic data in the so-called Porod region^37^, leading to a Porod slope value. Thus, fits were performed in the $Q$ region between 3.2 nm^−1^ and 4.5 nm^−1^ and reported for both samples in Fig. 4C. In contrast to the fractal slope, the Porod slope calculation required hexane subtraction (Figure S6 and Figure S7) due to the vicinity between the Porod and hexane scattering $Q$ region. This was also reported in the literature for a SCD aerogel in comparison to a gel filled with solvent, showing an almost parallel scattering signal in the low and intermediate, and a rising influence in the high $Q$ region^38^. In fact, the influence of solvent scattering becomes less and less relevant while drying and Porod slope values converge when the hexane content is negligible. Moreover, at early evolution stages, fit results can be severely biased if no hexane subtraction is considered (Figure S2). The UN sample shows an increase of the slope from − 3.24 ± 0.01 (UN1) to − 3.07 ± 0.01 (UN3), afterwards dropping to − 3.56 ± 0.01 (UN7) followed by a small increase to − 3.39 ± 0.01. Contrary, the TM sample shows constant values of around − 3.40 ± 0.01 (TM1 and TM3) for approximately four hours. Later, the slope starts to decrease, dropping from − 3.49 ± 0.01 (TM5) to − 4.00 ± 0.01 (TM7), finally reaching − 4.05 ± 0.01 (TM8). This sharp decrease in slope could be correlated with the SBE: Fig. 5 summarizes these findings (see also Table S1). No change in slope for the TM sample was observed until the sample was completely shrunk. The SBE and the expansion of the material correlated with a decrease of the slope, reaching ca. − 4 at the end, indicating smooth surfaces. Contrary, a slope of − 3.4 implies fractally rough surfaces for the TM sample at the start and the UN sample at the start and the end^20^. Similar values are also reported in the literature for silica gels dried at different temperatures^39^. Since a Porod slope of − 4 was also verified for supercritically dried aerogels in the past^40^, the effect of surface modification on the surface roughness should be negligible. Thus, the fractally rough surfaces of the UN sample might be affected by fractures and micropore permeation^41^. Furthermore, the network might consist of polymeric clusters instead of dense particles^42^.

Additional measurements were conducted on dried reference samples to compare with the UN8 and TM8 measurements of the in-situ experiments (Fig. 4D and Note S3). These samples were dried three to seven days prior to the synchrotron experiments and originated from the same batch of the synthesis. Almost no differences in the scattering curve can be recognized comparing the unmodified samples (blue and cyan lines). Similarly, the modified samples (red, orange and yellow lines) showed only slight differences in the $Q$ region around 10 nm^−1^.

This might indicate that either (i) the in-situ sample had a small residual amount of hexane inside the network, (ii) the room temperatures for drying differed slightly, leading to a small change in the silica structure, or (iii) pore sizes keep recovering very slowly after complete drying, meaning the structure might have a relaxation effect after complete drying.

## Conclusions

For understanding the structural changes in silica aerogels during the drying process with a special focus on the SBE phenomenon, monolithic gels with high specific surface areas (up to 802 m^2^g^−1^) were successfully synthesized by APD and investigated by in-situ SAXS during the drying of the aerogels. The change in the hexane content during the drying process was estimated from the scattering data, which revealed that hexane remained inside the material at maximum shrinkage, but almost none after the SBE. The collapse of pores due to drying and the re-expansion in surface-modified samples because of the SBE was visible in the scattering curves, suggesting a similar shape of the constituents’ elementary units of the network at the start and end of the experiment. Both, unmodified and surface-modified samples, show a porous fractal nanostructure, which partially collapses during hexane evaporation and is only recovered in the surface-modified samples during the SBE. Evaluation of the fractal slope allowed to correlate the nanostructure with macroscopic sample characteristics. Samples that recover their original geometry seem to regain the initial fractal slope values, making this feature a distinct measure for complete SBE.

Furthermore, the drying velocity was significantly slowed down by surface modification. The results of the Porod slope calculation during hexane evaporation found that the SBE in surface-modified samples is accompanied by a sudden change from a fractally rough to a smooth surface, which might be interpreted as a recovery of collapsed small pores below a few nanometers. Since the nanostructure determines most potential applications of aerogels, these findings may not only contribute to a basic understanding of the structural changes during the SBE, but may also help to compose tailored aerogels with specific functionality.

## Experimental

### Materials

Tetraethyl-orthosilicate (TEOS, reagent grade 98%; Merck), ethanol (≥ 99.5%, Ph.Eur., reinst; Roth) deionized water, hydrochloric acid (37%; Merck), and ammonium hydroxide solution in water (25%; Merck) were used as supplied. Furthermore, hexane (n-hexane, > 99%; Carl Roth) and trimethylchlorosilane (TMCS, purified by redistillation > 99%; Merck) were used as received.

### Sample preparation

Ambient pressure dried silica aerogels and unmodified gels were prepared by sol–gel method, similar to that reported by Wei et al.^43^. Therefore, the silica precursor TEOS (20.833 g) was mixed with ethanol (8.759 ml), an ethanol/37% hydrochloric acid solution (8.759 ml/2.08 µl), and distilled water (1.801 ml) to form a sol. After 90 min, further ethanol (37.003 ml), and 25% ammonium hydroxide solution in water (31.6 µl in 4.683 ml) were added to produce 6 g of SiO_2_ (i.e., 8.5 wt% of the final gel). The solution was transferred to smaller custom-made closed Teflon molds and left to gel and age for 24 h at 50 °C. The cuboid wetgels were washed with excess ethanol for 24 h at 50 °C. Afterwards, the ethanol was subsequently exchanged by mixtures of ethanol and hexane (25 vol%/75 vol%, 50 vol%/50 vol%, 75 vol%/25 vol%), and finally rinsed four times with pure hexane for 24 h, each. Thereafter, half of the samples were modified using first an excess of twice a 3 vol% and then twice a 6 vol% solution of TMCS in hexane, thus performing the surface modification in four steps. Hydrochloric acid and TMCS residues were removed by washing with an excess amount of hexane. The TMCS surface modified (denoted: TM) and unmodified (denoted as: UN) samples used for in-situ measurements were stored in an excess amount of hexane at room temperature. In-situ samples were dried during the measurements. Afterwards, samples were left to dry up to five days after experiments were completed.

### Characterization

**Figure 6:**
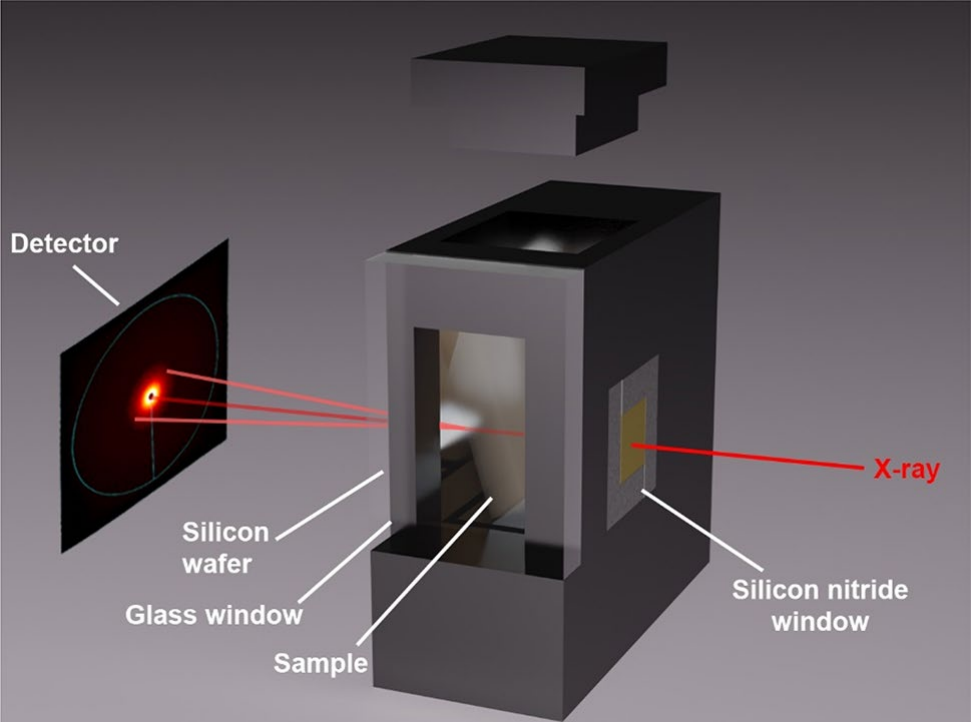
Rendering (Blender v2.90.1 https://www.blender.org/) of the in-situ measurement cell and the x-ray pathway through it. The measurement cell consists of anodized aluminium and is sealed with a silicon wafer, a silicon nitride window, a museum-glass window and an aluminium lid.

The in-situ small-angle/wide-angle X-ray scattering measurements were performed at the µSpot beamline of the Max Planck Institute of Colloids and Interfaces at the BESSY II (Helmholtz Zentrum für Materialien und Energie, Berlin, Germany)^44^. The samples soaked in hexane were transferred to measurement cells (Fig. 6) consisting of anodized aluminium. The measurement cells were sealed off with a silicon wafer and a silicon nitride window in the X-ray beam direction; and a piece of museum glass (glass window) on the side to allow imaging of the samples while minimizing reflections. A digital microscope camera (TOOLKRAFT USB microscope, 5 MP) was used for collecting pictures of the sample every second. Once the samples were transferred into the measuring cell, the aluminum lid was attached to partially close off the top of the cell to allow for slow drying. Alternating an empty cell, as well as each of the two measuring cells were measured at three different heights. The measurements were carried out using a B4C/Mo Multilayer (2 nm period) monochromator and an energy of 15 keV. A sequence of pinholes was used to select 100 × 100 µm^2^ spotsize. Scattering data were collected by an Eiger 9 M detector with 75 × 75 µm^2^ pixel area (Figure S1). Further data processing was done using the directly programmable data analysis kit (DPDAK)^45^. Data were normalized over primary beam intensity and background subtracted. The latter was evaluated by measuring diffraction patterns through the silicon nitride and silicon wafer windows. Diffraction patterns were radially integrated and the scattered intensity $I(Q)$ was calculated as a function of the momentum transfer $Q$, defined as

2 $$Q=\frac{4\pi }{\lambda }\mathrm{sin}\left(\frac{\theta }{2}\right)$$

with $\lambda$ and $\theta$ the photon wavelength and the scattering angle, respectively.

The sample to detector distance was set to 333.52 mm in order to access to a $Q$ region between 0.12 and 31.18 nm^−1^. Data were evaluated and fitted by using the DPDAK Peak Fit plugin^45^, as well as Python scipy stats library^46^. The reported errors were rounded to two decimal points, and represent statistical errors associated to the fitting parameters. The relative size of the samples was estimated by measuring an area inside the photographs with ImageJ v1.53 k.

The surface morphology of the ambient pressure dried gels was visualized by means of scanning electron microscopy (SEM) using a LEO Gemini 1530 (Zeiss, Germany) at 3 kV, 30 µm aperture with an Inlens detector.

The chemical composition and confirmation of the surface modification via silylation agent were measured with attenuated total reflection Fourier-Transform Infrared Spectroscopy (ATR-FTIR) using a Bruker Optik GmbH Vertex 70. For this purpose, samples were ground to get a fine powder distribution.

Isotherms were recorded by a Quadrasorb of Quantachrome (by 3P instruments) using nitrogen sorption at − 195.85 °C. Samples were cut into smaller pieces to fit into the measurement cell. Prior to measurement, the samples were degassed and dried at 200 °C for 12 h and the measurement cell was cooled with liquid nitrogen. The specific surface area was determined using BET (Brunauer–Emmett–Teller equation). Since this approach is only applicable for samples without microporosity, the Rouquerol-Plot was used to alter the pressure range following the recommendation of IUPAC^29^.

## Supplementary Information

Supplementary Information.

## Data Availability

Additional findings and evaluations are accessible in the supporting information. Further data is available from the corresponding authors upon reasonable request.
