# Supplementary material for: Springback effect and structural features during the drying of silica aerogels tracked by in-situ synchrotron X-ray scattering
Source: Sci Rep. 2022 May 9;12:7537. doi: 10.1038/s41598-022-11127-6 (PMC9085844; doi:10.1038/s41598-022-11127-6)
Supplement: Supplementary file 1 — Supplementary Information. [file 41598_2022_11127_MOESM1_ESM.docx]

Supplementary information

Springback effect and structural features during the drying of silica aerogels tracked by *in-situ* synchrotron X-ray scattering

Fabian Zemke^1*^, Ernesto Scoppola^2^, Ulla Simon^1^, Maged F. Bekheet^1^, Wolfgang Wagermaier^2*^, Aleksander Gurlo^1^

^1^Technische Universität Berlin, Faculty III Process Sciences, Institute of Materials Science and Technology, Chair of Advanced Ceramic Materials, Straße des 17. Juni 135, 10623 Berlin, Germany

^2^Max Planck Institute of Colloids and Interfaces, Department of Biomaterials, Am Mühlenberg 1, 14476 Potsdam, Germany

*email: f.zemke@tu-berlin.de, wolfgang.wagermaier@mpikg.mpg.de

**Overview supplementary information:**

- **Note S1**: Fractal slope calculation.
- **Note S2**: Calculation of the hexane content.
- **Note S3**: Reproducibility of *in-situ* measurements.
- **Figure S1**: Overview of the setup used for the *in-situ* X-ray scattering experiments, consisting of a beam stop, Eiger 9M detector, Cyberstar detector, filter wheel, fast shutter, slits, pinholes, ionization chamber and sample holder.
- **Figure S2**: Slope fits for the Porod slope for the duration of the experiment of UN (A, green/blue) and TM (B, yellow/red) without the correction of the hexane peak.
- **Figure S3**: Lorentzian peak fits of the unmodified sample UN for the duration of the experiment. The area was calculated from the full-width at half-maximum and intensity at a fixed position of $Q=13.73$ nm^‑1^ (LO1, cyan) and $Q=16.80$ nm^‑1^ (LO2, blue).
- **Figure S4**: Lorentzian peak fits of the surface modified sample TM for the duration of the experiment. The area was calculated from the full-width at half-maximum and intensity at a fixed position of $Q=13.73$ nm^‑1^ (LO1, orange) and $Q=16.80$ nm^‑1^ (LO2, red).
- **Figure S5**: The percentage of hexane in the UN (green/blue) and TM (yellow/red) samples over time calculated from the decoupling of the peak in the region 13.73 nm^‑1^ for the Lorentzian (“Lorentz”) and hexane scattering profile subtraction (“Subtr.”) approach.
- **Figure S6**: Small-angle X-ray scattering (SAXS) curves of the unmodified UN sample corrected for hexane content in the sample over time.
- **Figure S7**: Small-angle X-ray scattering (SAXS) diagrams of the modified TM sample corrected for hexane content in the sample over time.
- **Figure S8**: A) Development of the fractal slope for different modified (red, orange) and unmodified (blue, cyan) samples over time. B) Evaluation of the Porod slope over time for different modified (red, orange) and unmodified (blue, cyan) samples.
- **Figure S9:** The percentage of hexane in the UN (blue) and TM (red) samples overplotted with the transmission (cyan and orange, respectively) over time.
- **Figure S10**: Digital images of the UN sample over time inside the *in-situ* measurement chamber.
- **Figure S11**: Digital images of the TM sample over time inside the *in-situ* measurement chamber.
- **Figure S12**: A), C) Azimuthal integration over Q for the TM sample of the first and last measurement, respectively; and B), D) Azimuthal integration over Q for the UN sample of the first and last measurement, respectively. Data shows isotropic diffraction signal along azimuthal direction.
- **Figure S13.** A) Nitrogen adsorption (Ads.) – desorption (Des.) isotherms of the dried TM sample (red) and B) Nitrogen adsorption (Ads) – desorption (Des.) isotherms of the dried UN sample (blue).
- **Table S1**: Overview of distinct features of the in-situ X-ray scattering experiment. The time, calculated hexane volume content, fractal slope, Porod slope (hexane content subtracted), as well as the qualitative state of drying are summarized for specific points of interest UN1‑UN8 and TM1‑TM8, showing the development during the experiment.

Note S1. Fractal slope calculation

The fractal slope was calculated in an intermediate *Q* range ($0.3\mathrm{nm}^{-1} \leq Q \leq2.6 \mathrm{nm}^{-1}$). For every measurement, a linear decay over a range of 0.6$\mathrm{nm}^{-1}$ in the double logarithmic scale of scattering profiles was extrapolated. Here, the slope was calculated by linear regression, moving the lower and upper limits by 0.02$\mathrm{nm}^{-1}$ increments. The *Q* range deviation was restricted to 0.2$\mathrm{nm}^{-1}$ between two subsequent measurement points. The slope value as a function of time corresponding to the best fit model minimizing the correlation coefficient, i.e., maximizing the $R^{2}$, were used.

Note S2. Calculation of the hexane content

Two different approaches were used to calculate the hexane evaporation from the scattering data. In a first attempt, data were modelled by assuming two Lorentzian curves and a linear background. The peak positions were fixed at 13.73 nm^‑1^ and 16.80 nm^‑1^ for hexane and silica, respectively. The full‑width at half‑maximum (FWHM) $w_{i}$, as well as the intensity $a_{i}$, were evaluated for the duration of the experiment, though $w_{i}$ was fixed for the hexane curve. The area $A_{i}$ of the two peaks was calculated by using:

| $A_{i}=\frac{\pi\cdot a_{i}\cdot w_{i}}{2}$ | Equation S1 |
| --- | --- |

Figure S3 and Figure S4 visualize the development of Lorentzian peak areas over time, and afterwards expressed as percentages, where 100 % was assumed for the first measurement point and 0 % for the last (Figure S5: Unmodified (UN) blue and Modified (TM) red).

For the second approach, the hexane scattering profile was brought into conjunction with the first measurement of UN and TM, by applying a factor of 0.42 and 0.52 respectively to match the intensities. Afterwards, Equation S1 was used to determine $\alpha(t)$ to match the $I\left( Q,t \right)$ at $Q=13.73$ nm^‑1^ and interpreted as percentages of hexane intensity (Figure S5: Unmodified (UN) cyan and Modified (TM) orange). Here, it was assumed that the maximum at this region is only influenced by a change in hexane content. Generally, the Lorentzian Fit approach seems to show a higher drying velocity in comparison to the second approach, showing ca. 42 % hexane intensity in comparison to 53 % at 3.7 h. As visualized in Figure S3 and Figure S4, the silica peak contribution did not seem to be constant, suggesting that the fitting approach with two Lorentzian distributions, provides more reliable results.

Note S3. Reproducibility of *in-situ* measurements

As visualized in Figure S8, different unmodified and modified samples were measured *in‑situ* and the fractal and Porod slopes were compared. Here, the results originated from different synchrotron experiments, different batches of syntheses and different batches of chemicals used. Nonetheless, the general steps for sol to gel transformation and surface modification were not altered. Though they show small differences in absolute values, the measurements of the two unmodified samples (blue and cyan) were very similar in terms of the development of the fractal and Porod slope.


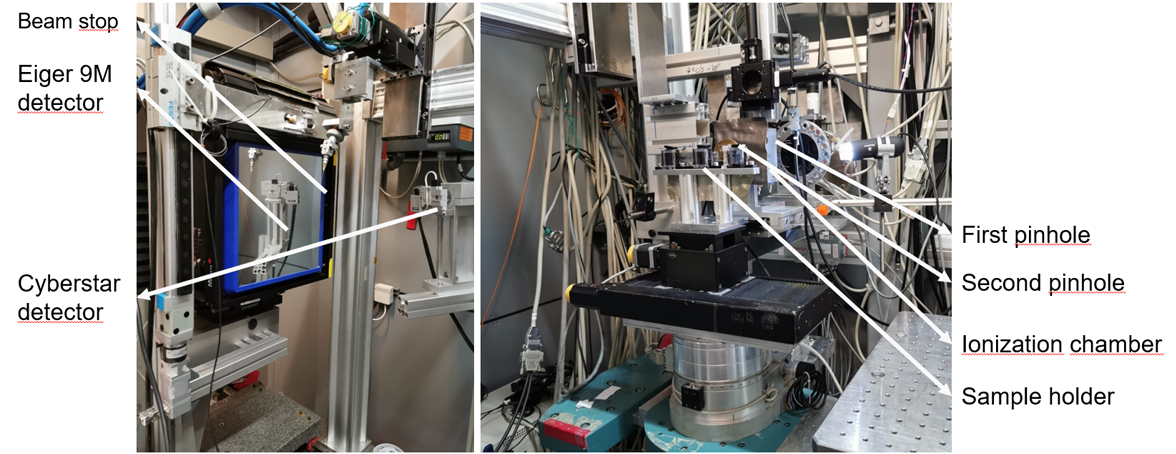


Figure S1. Overview of the setup used for the *in-situ* X-ray scattering experiments, consisting of a beam stop, Eiger 9M detector, Cyberstar detector, filter wheel, fast shutter, slits, pinholes, ionization chamber and sample holder.


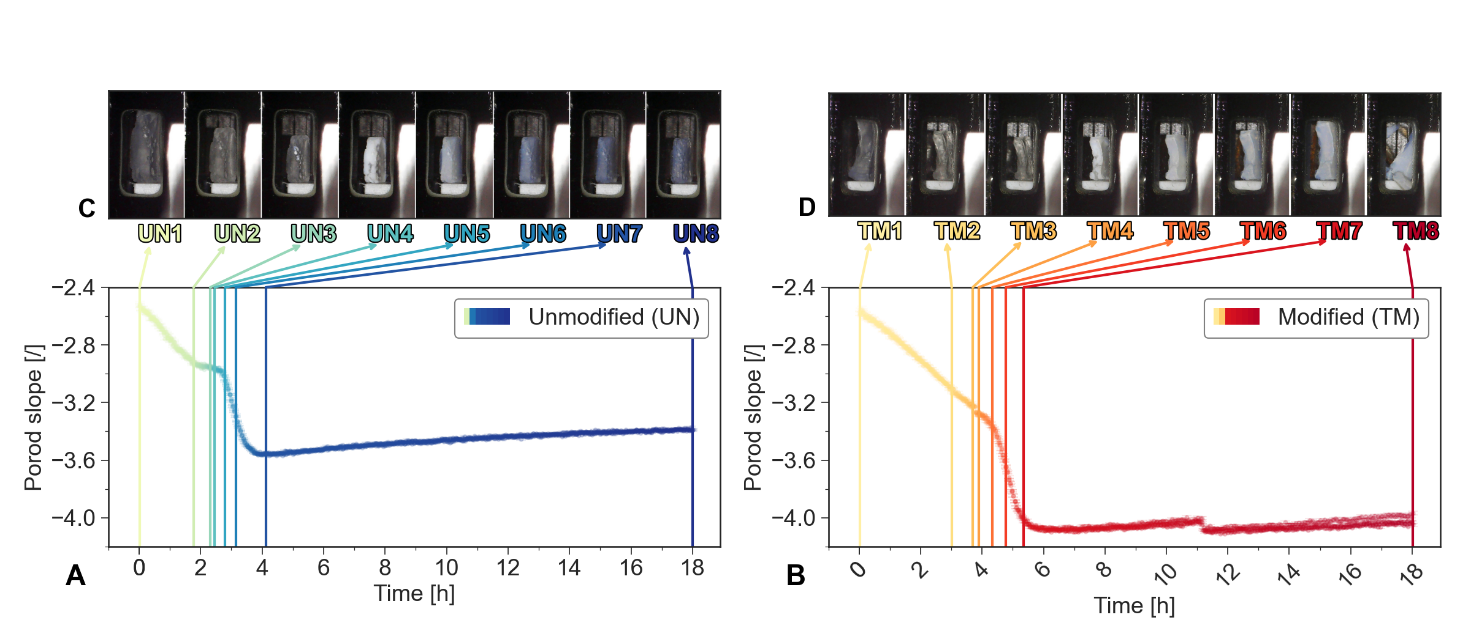


Figure S2. Slope fits for the Porod slope for the duration of the experiment of UN (A, green/blue) and TM (B, yellow/red) without the correction of the hexane peak.


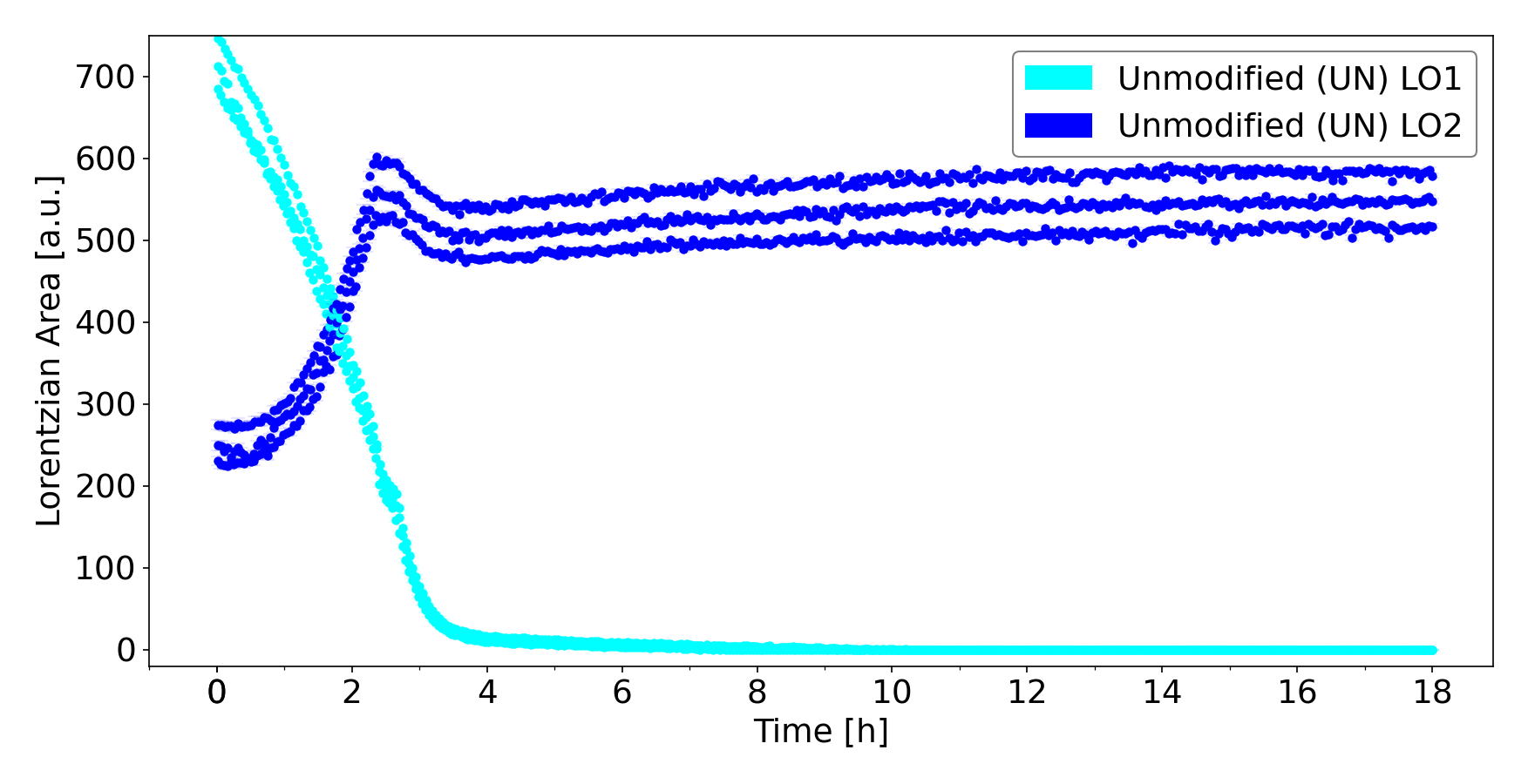


Figure S3. Lorentzian peak fits of the unmodified sample UN for the duration of the experiment. The area was calculated from the full-width at half-maximum and intensity at a fixed position of $Q=13.73$ nm^‑1^ (LO1, cyan) and $Q=16.80$ nm^‑1^ (LO2, blue).


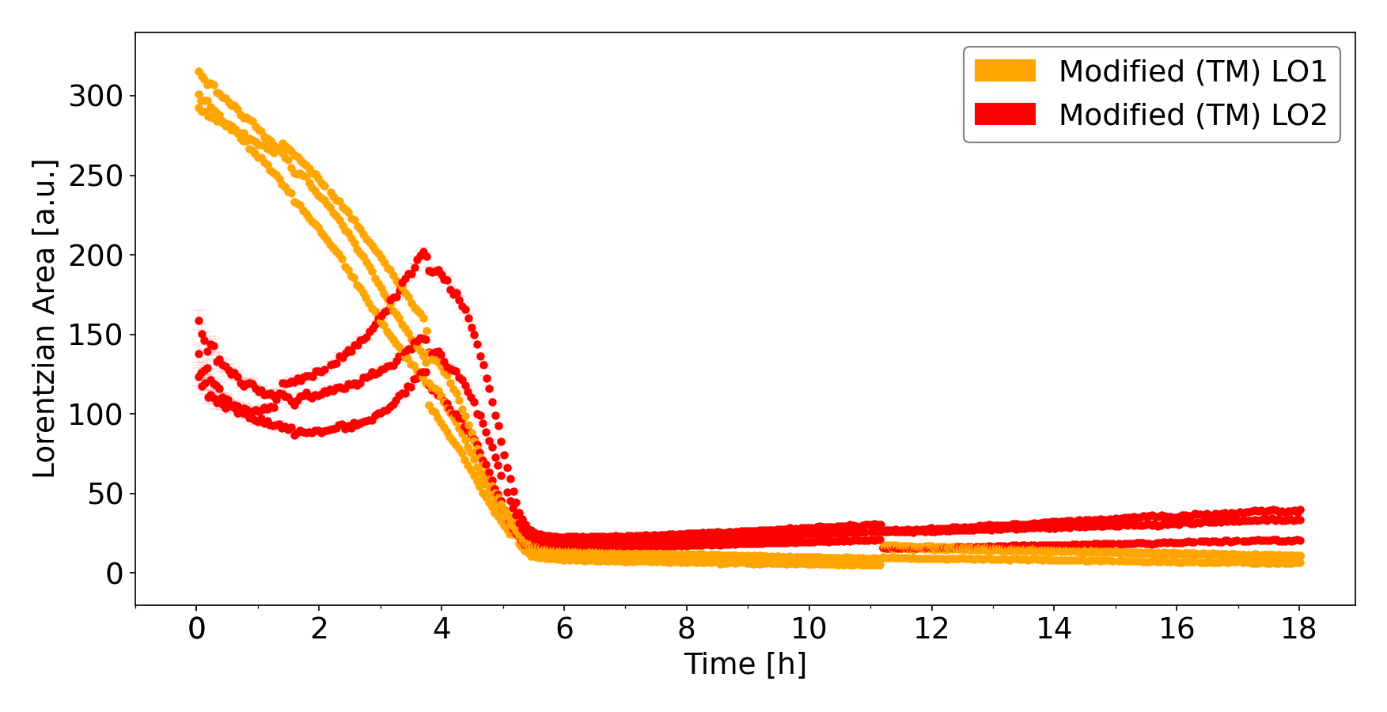


Figure S4. Lorentzian peak fits of the surface modified sample TM for the duration of the experiment. The area was calculated from the full-width at half-maximum and intensity at a fixed position of $Q=13.73$ nm^‑1^ (LO1, orange) and $Q=16.80$ nm^‑1^ (LO2, red).


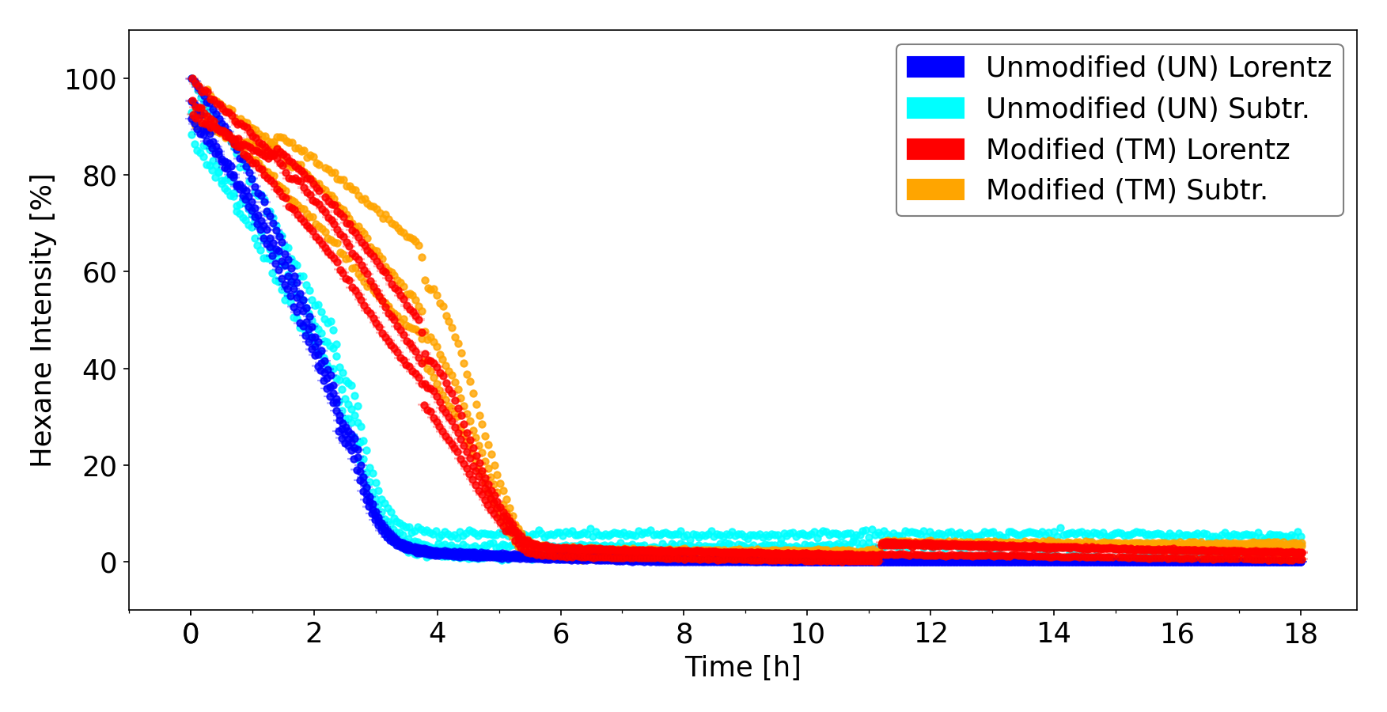


Figure S5. The percentage of hexane in the UN (green/blue) and TM (yellow/red) samples over time calculated from the decoupling of the peak in the region 13.73 nm^‑1^ for the Lorentzian (“Lorentz”) and hexane scattering profile subtraction (“Subtr.”) approach.


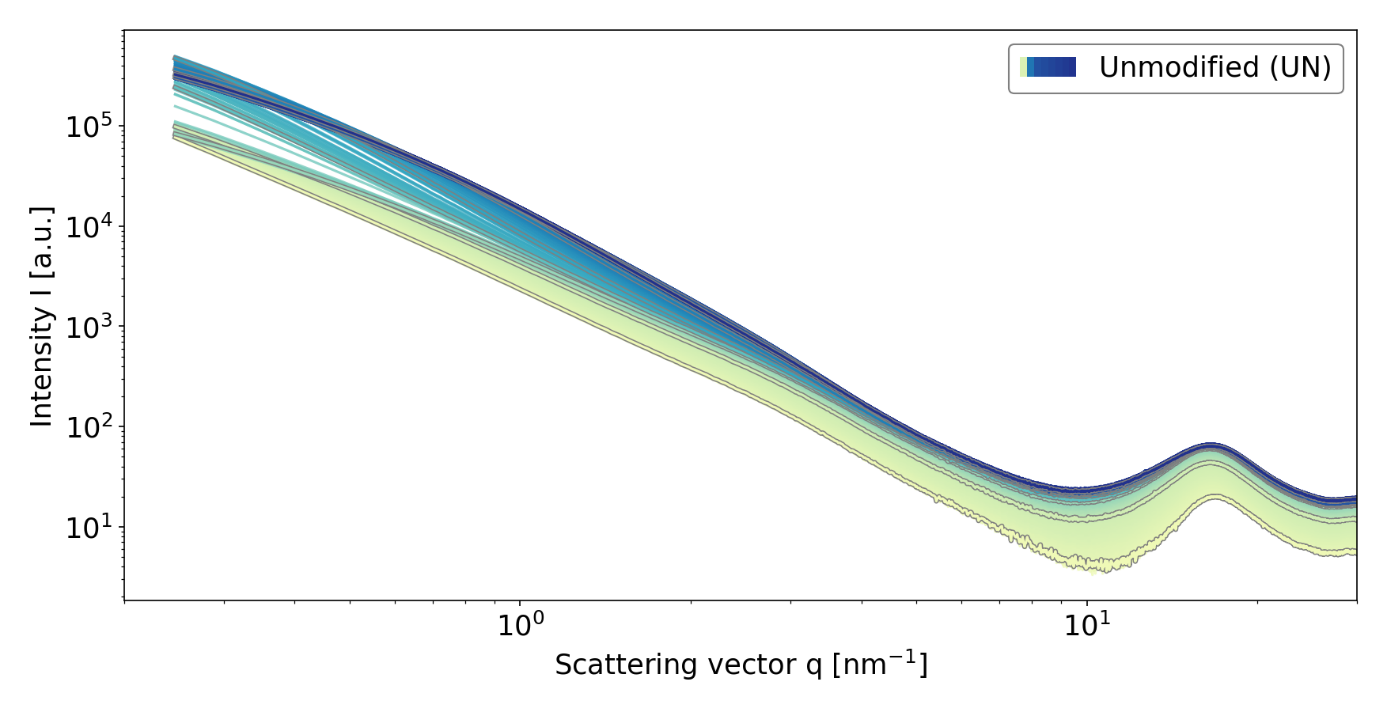


Figure S6. Small-angle X-ray scattering (SAXS) curves of the unmodified UN sample corrected for hexane content in the sample over time.


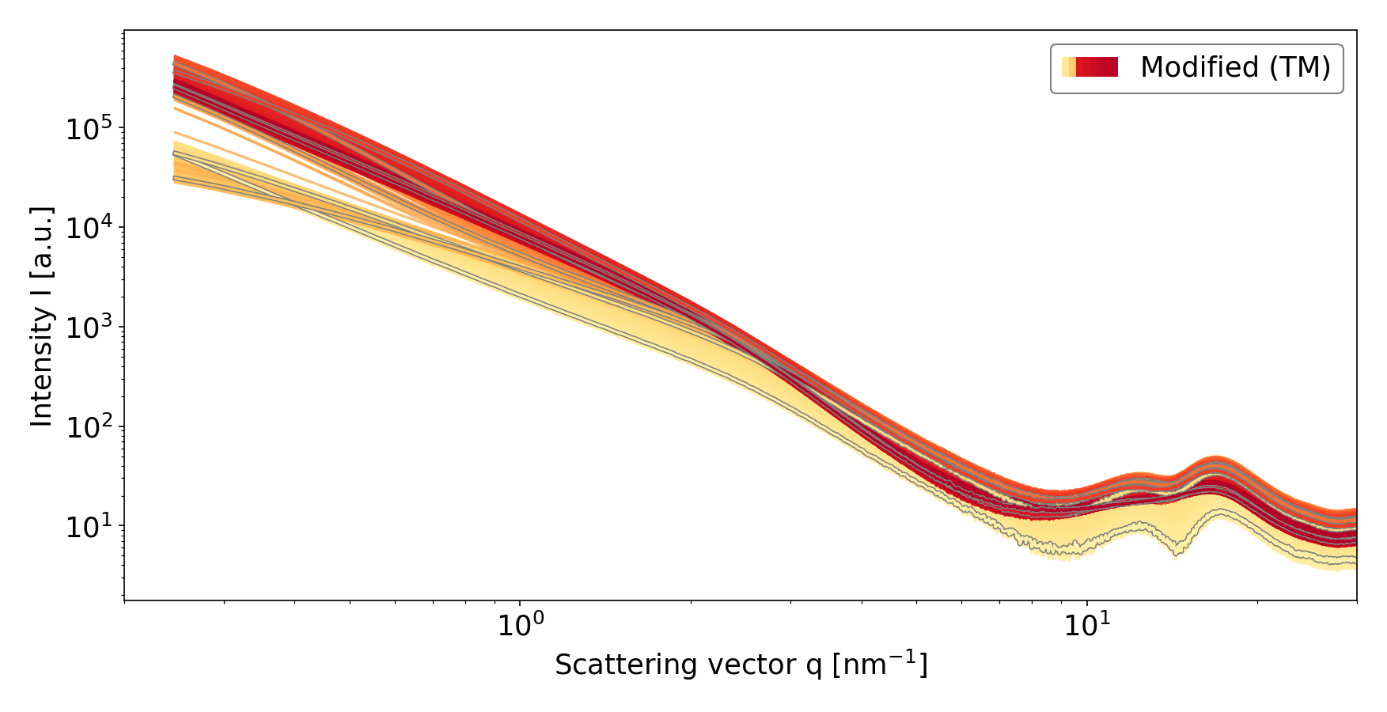


Figure S7. Small-angle X-ray scattering (SAXS) diagrams of the modified TM sample corrected for hexane content in the sample over time.


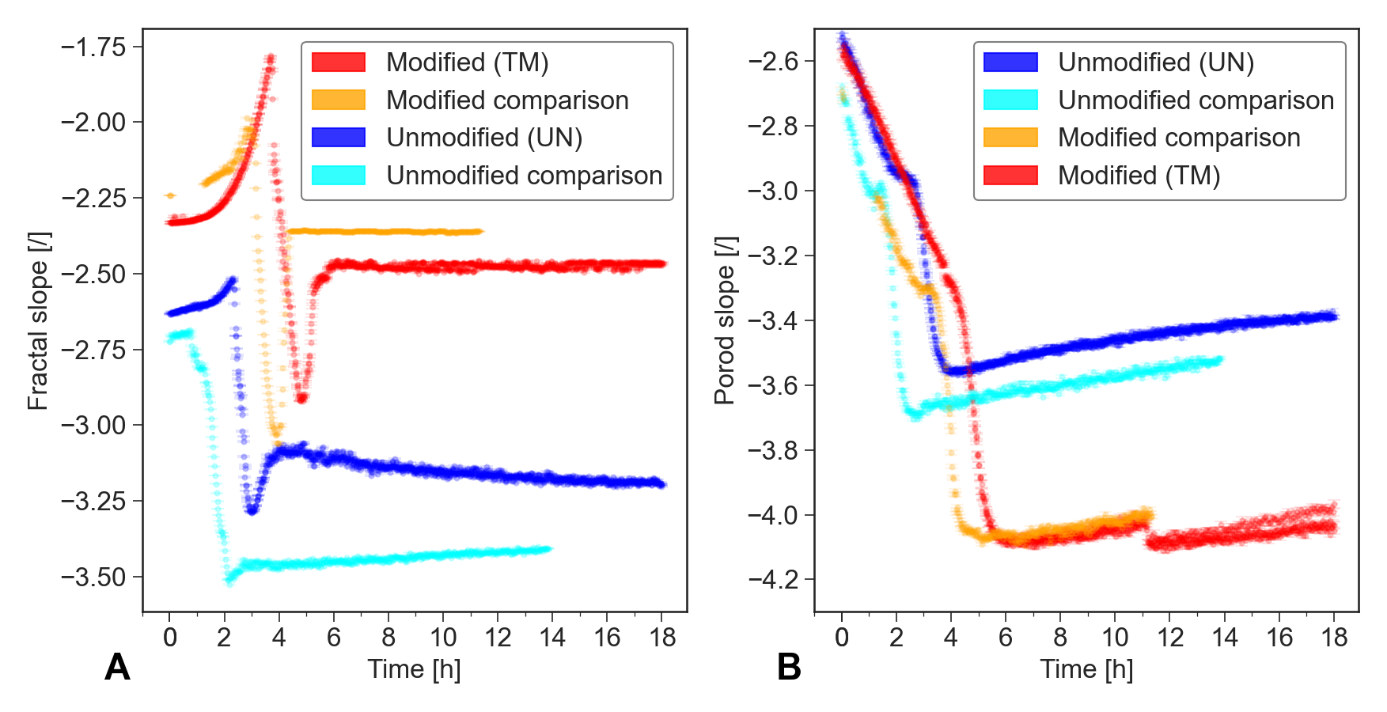


Figure S8. A) Development of the fractal slope for different modified (red, orange) and unmodified (blue, cyan) samples over time. B) Evaluation of the Porod slope over time for different modified (red, orange) and unmodified (blue, cyan) samples.


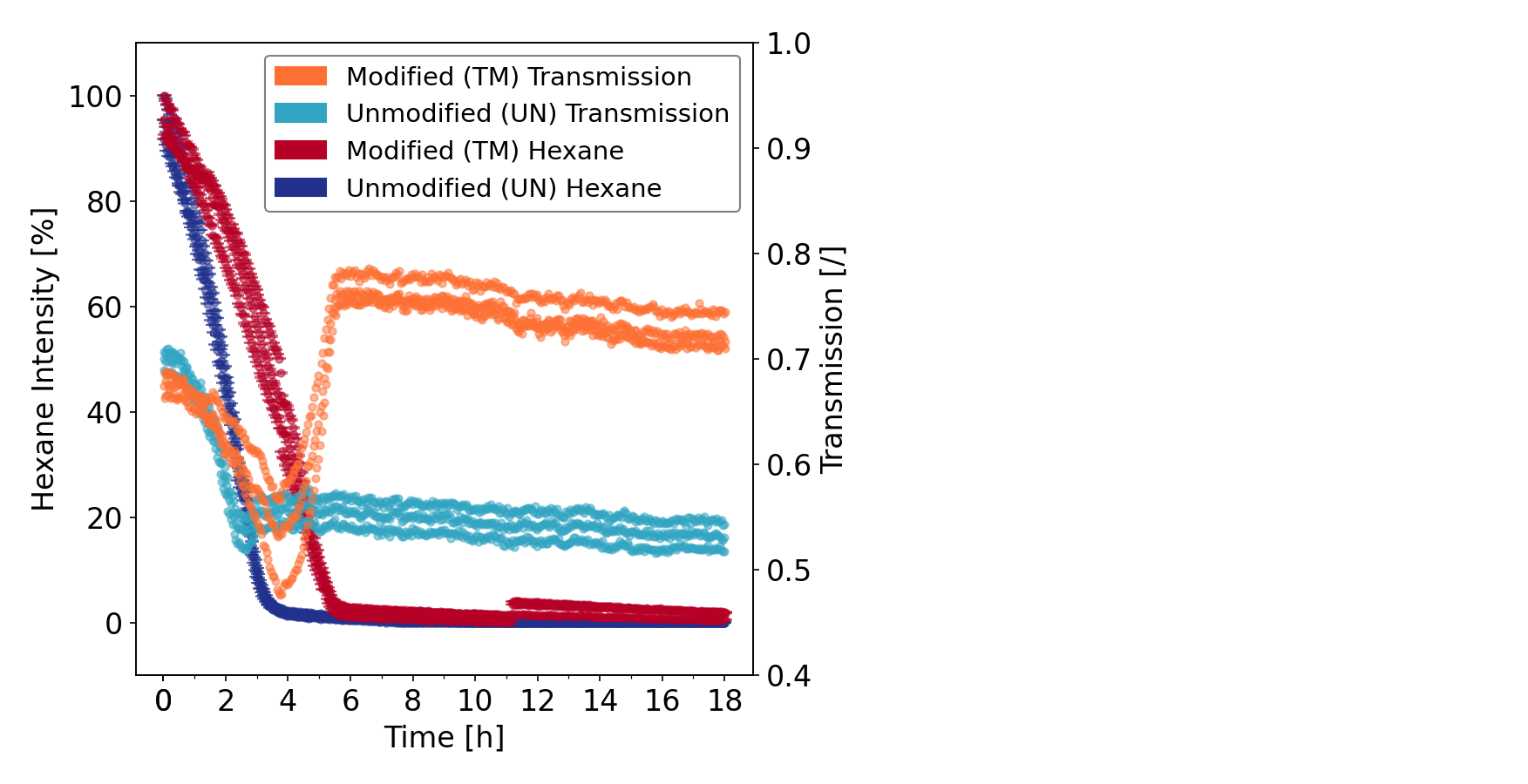


Figure S9. The percentage of hexane in the UN (blue) and TM (red) samples overplotted with the transmission (cyan and orange, respectively) over time.


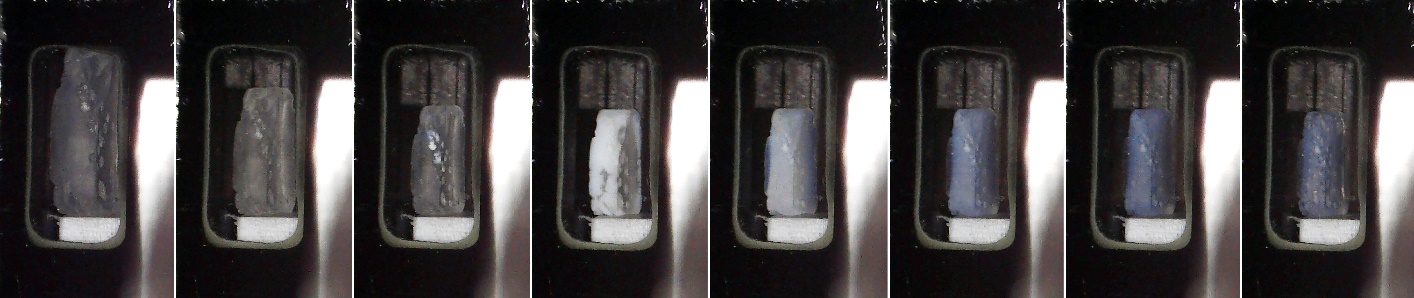


Figure S10. Digital images of the UN sample over time inside the *in-situ* measurement chamber.


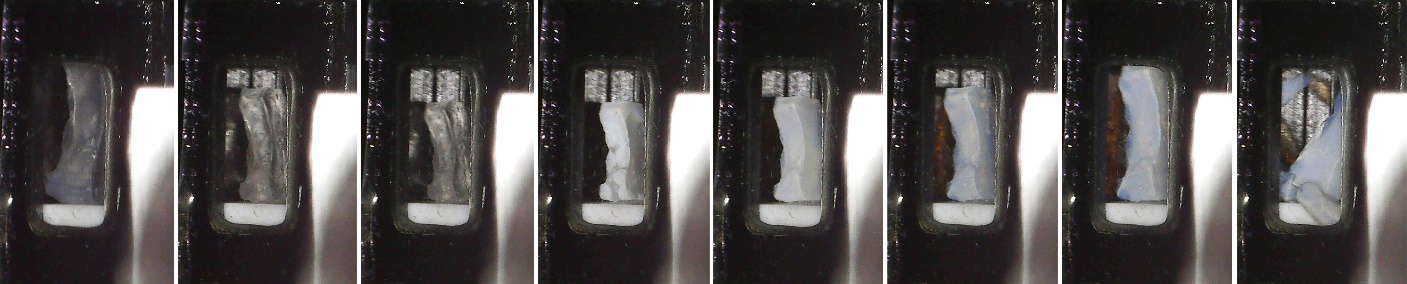


Figure S11. Digital images of the TM sample over time inside the *in-situ* measurement chamber.


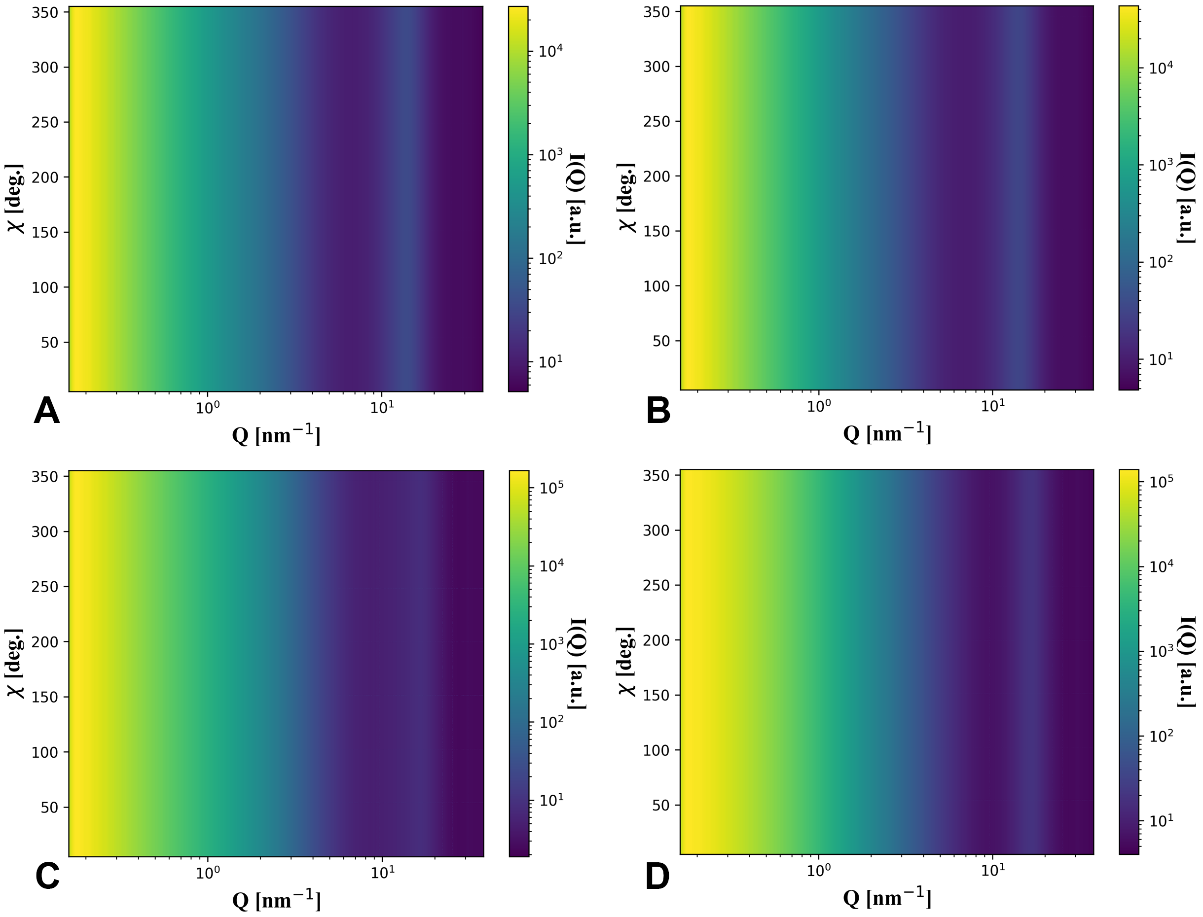


Figure S12. A), C) Azimuthal integration over Q for the TM sample of the first and last measurement, respectively; and B), D) Azimuthal integration over Q for the UN sample of the first and last measurement, respectively. Data shows isotropic diffraction signal along azimuthal direction.


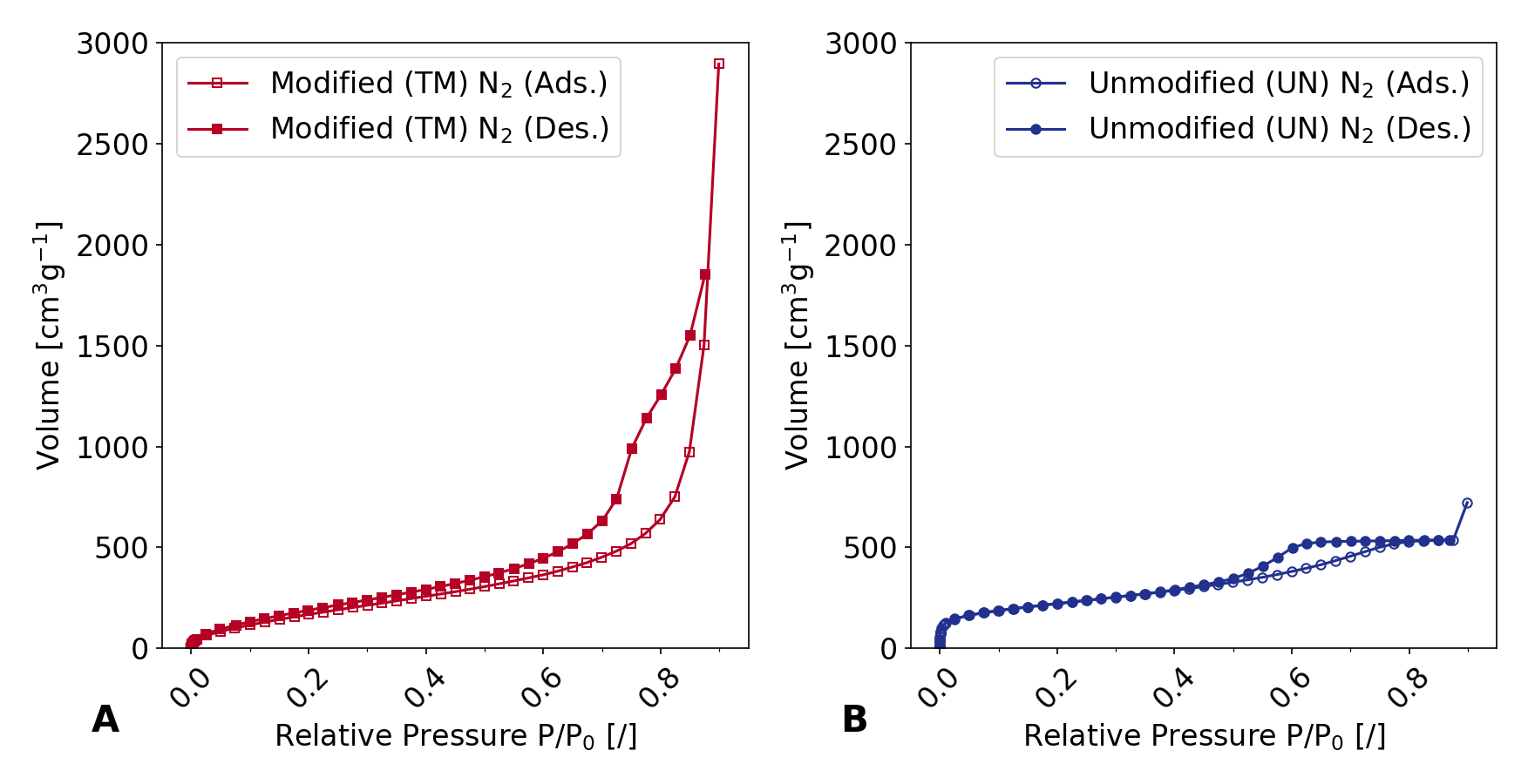


Figure S13. A) Nitrogen adsorption (Ads.) – desorption (Des.) isotherms of the dried TM sample (red) and B) Nitrogen adsorption (Ads) – desorption (Des.) isotherms of the dried UN sample (blue).

Table S1. Overview of distinct features of the in-situ X-ray scattering experiment. The time, calculated hexane volume content, fractal slope, Porod slope (hexane content subtracted), as well as the qualitative state of drying are summarized for specific points of interest UN1‑UN8 and TM1‑TM8, showing the development during the experiment.

|  |  | 1 | 2 | 3 | 4 | 5 | 6 | 7 | 8 |
| --- | --- | --- | --- | --- | --- | --- | --- | --- | --- |
| State of drying | UN | Wet gel | Shrinking | Max. shrink. | Max. shrink. | Max. shrink. | Max. shrink. | Max. shrink. | Max. shrink. |
|  | TM | Wet gel | Shrinking | Max. shrink. | Start reexpans. | SBE | SBE | Aerogel | Aerogel |
| Appearance | UN | Transp. | Transp. | Transp. | White-opaque | Blue-opaque | Translucent | Blue-transp. | blue-transp. |
|  | TM | Transp. | Transp. | Transp. | White-opaque | White-opaque | White-opaque | Blue-opaque | Blue-opaque |
| Time [s] | UN | 45 | 6366 | 8311 | 8838 | 10068 | 11301 | 14820 | 64799 |
|  | TM | 103 | 10830 | 13307 | 14023 | 15595 | 17181 | 19298 | 64857 |
| Hexane [vol%] | UN | 95.36  ±0.07 | 53.24  ±0.06 | 34.88  ±0.11 | 27.54  ±0.06 | 16.35  ±0.05 | 6.50  ±0.05 | 1.74  ±0.04 | 0.00  ±0.03 |
|  | TM | 100.00  ±0.11 | 56.06  ±0.10 | 42.27  ±0.11 | 35.79  ±0.11 | 26.72  ±0.10 | 15.17  ±0.08 | 7.01  ±0.06 | 0.51  ±0.07 |
| Fractal slope [/] | UN | ‑2.63  ±0.01 | ‑2.59  ±0.01 | ‑2.52  ±0.01 | ‑2.75  ±0.01 | ‑3.20  ±0.01 | ‑3.27  ±0.01 | ‑3.09  ±0.01 | ‑3.20  ±0.01 |
|  | TM | ‑2.33  ±0.01 | ‑2.06  ±0.01 | ‑1.79  ±0.01 | ‑2.15  ±0.01 | ‑2.57  ±0.01 | ‑2.92  ±0.01 | ‑2.54  ±0.01 | ‑2.47  ±0.01 |
| Porod  slope [/] | UN | ‑3.24  ±0.01 | ‑3.18  ±0.01 | ‑3.07  ±0.01 | ‑3.05  ±0.01 | ‑3.10  ±0.01 | ‑3.32  ±0.01 | ‑3.56  ±0.01 | ‑3.39  ±0.01 |
|  | TM | ‑3.40  ±0.01 | ‑3.42  ±0.01 | ‑3.40  ±0.01 | ‑3.42  ±0.01 | ‑3.49  ±0.01 | ‑3.73  ±0.01 | ‑4.00  ±0.01 | ‑4.05  ±0.01 |
